# Analysis of coexisting pathogens in nasopharyngeal swabs from COVID-19

**DOI:** 10.3389/fcimb.2023.1140548

**Published:** 2023-06-22

**Authors:** Zhan Gao, Lisong Yu, Ling Cao, Meng Yang, Yuhui Li, Yue Lan, Ruixiang Tang, Yang Huang, Guangxin Luan, Yingfen Liu, Hailin Yu, Ling Jian, Yi Zha, Zhenxin Fan, Yujiao Bai, Mei Luo, Miao He, Shanshan Deng

**Affiliations:** ^1^ Institute of Blood Transfusion, Chinese Academy of Medical Sciences, Chengdu, China; ^2^ Information Institute, Shanghai Municipal Center for Disease Control and Prevention, Shanghai, China; ^3^ Department of Information Technology, Assumption University, Bangkok, Thailand; ^4^ Department of Laboratory Medicine, Public Health and Clinical Center of Chengdu, Chengdu, China; ^5^ Non-Coding RNA and Drug Discovery KeyLaboratory of Sichuan Province, Chengdu Medical College, Chengdu, China; ^6^ Human Leukocyte Antigen (HLA) Typing Laboratory, Blood Center of Shaanxi Province, Institute of Xi’an Blood Bank, Xi’an, China; ^7^ College of Life Sciences, Sichuan University, Chengdu, China

**Keywords:** SARS-CoV-2, co-infection, respiratory pathogens, high-throughput sequencing, metagenomics

## Abstract

**Background:**

The impact of COVID-19 on the world is still ongoing, and it is currently under regular management. Although most infected people have flu-like symptoms and can cure themselves, coexisting pathogens in COVID-19 patients should not be taken lightly. The present study sought to investigate the coexisting pathogens in SARS-CoV-2 infected patients and identify the variety and abundance of dangerous microbes to guide treatment strategies with a better understanding of the untested factors.

**Methods:**

We extracted total DNA and RNA in COVID-19 patient specimens from nasopharyngeal swabs to construct a metagenomic library and utilize Next Generation Sequencing (NGS) to discover chief bacteria, fungi, and viruses in the body of patients. High-throughput sequencing data from Illumina Hiseq 4000 were analyzed using Krona taxonomic methodology for species diversity.

**Results:**

We studied 56 samples to detect SARS-CoV-2 and other pathogens and analyzed the species diversity and community composition of these samples after sequencing. Our results showed some threatening pathogens such as *Mycoplasma pneumoniae*, *Klebsiella pneumoniae*, *Streptococcus pneumoniae*, and some previously reported pathogens. SARS-CoV-2 combined with bacterial infection is more common. The results of heat map analysis showed that the abundance of bacteria was mostly more than 1000 and that of viruses was generally less than 500. The pathogens most likely to cause SARS-CoV-2 coinfection or superinfection include *Streptococcus pneumoniae*, *Haemophilus influenzae*, *Staphylococcus aureus*, *Klebsiella pneumoniae*, and *Human gammaherpesvirus 4*.

**Conclusions:**

The current coinfection and superinfection status is not optimistic. Bacteria are the major threat group that increases the risk of complications and death in COVID-19 patients and attention should be paid to the use and control of antibiotics. Our study investigated the main types of respiratory pathogens prone to coexisting or superinfection in COVID-19 patients, which is valuable for identifying and treating SARS-CoV-2.

## Introduction

Globally, as of December 2022, over 650 million confirmed cases of COVID-19, including 6.65 million deaths (WHO, 2020). SARS-CoV-2 is mainly transmitted by respiratory droplets and is usually characterized by fever, cough, fatigue, and dyspnea after infection (Xie et al., 2021). Because of the similarity with the clinical symptoms of other respiratory tract infections, especially bacterial infections, the coexisting pathogens in COVID-19 patients should not be ignored in prevention and treatment (Morris et al., 2017; Alshaikh et al., 2022). Elderly patients with underlying medical conditions are at risk of SARS-CoV-2 infection (Chen et al., 2021). Due to the relatively low immune function, these patients are more likely to have coinfection and secondary infection.

The widespread presence of pathogens in the respiratory tract is not conducive to rehabilitating COVID-19 patients. Secondary infection in COVID-19 patients is a key risk factor affecting disease severity and mortality (Mirzaei et al., 2020). Studies have shown that about 1/5 of COVID-19 patients require hospitalization due to clinical complications, including coinfection, hyperinflammatory response, or tissue pneumonia (Musuuza et al., 2021). In addition, although the prevalence of bacterial coinfection in infected patients is relatively low, the widespread practical use of antibiotics in hospital and community environments poses new challenges in managing antibiotics (Rothe et al., 2021). Distinguishing between viruses and secondary or concomitant bacteria and fungi infections remains challenging for clinicians. This diagnostic uncertainty leads to blind and excessive dependence on antibiotics in COVID-19 patients. It triggers the potential for adverse reactions related to bacterial resistance that may be caused by excessive use of antibiotics. In addition, the widespread use of empirical antibiotics, corticosteroids, and inflammatory inhibitors may mask the underlying symptoms of infection and lead to delays and inadequate diagnosis of secondary infections (Stasi et al., 2020). The co-infectious pathogens that have been reported include *Human Metapneumovirus* (Touzard-Romo et al., 2013), *Influenza A virus* (Wu et al., 2020), *Legionella* sp (Arashiro et al., 2020)., and *HIV* (Zhu et al., 2020).

The impact of SARS-CoV-2 infection on the nasopharyngeal microbiome has not yet been well described. As is well known, the upper respiratory microbiota located in the human nasopharynx is a key guardian of respiratory health and can help prevent or resist the invasion of easily infectious respiratory pathogens (Gao et al., 2014). The various microbial species in the upper respiratory tract microbiota form their unique microbial environment and in most cases, exist in a harmless symbiotic form. Some of these microbial species also belong to opportunistic pathogens, which can become pathogenic when the nasopharyngeal microbiota is imbalanced (de Steenhuijsen Piters et al., 2016). Most of the previously published studies only focused on SARS-CoV-2, ignoring other pathogens coexisting with SARS-CoV-2.

Examining co-exiting pathogens is beneficial to better understanding microbes in SARS-CoV-2 infected patients and to guide treatment strategies against those coexisted pathogens. In the present study, we conducted a metagenomic analysis of pathogens coexisting in the respiratory tract of SARS-CoV-2 infected patients, aiming to identify threatening pathogens and improve specific treatment plans for COVID-19 patients, which will help optimize treatment and minimize the negative impact of excessive use of antibiotics.

## Methods

**Figure 1:**
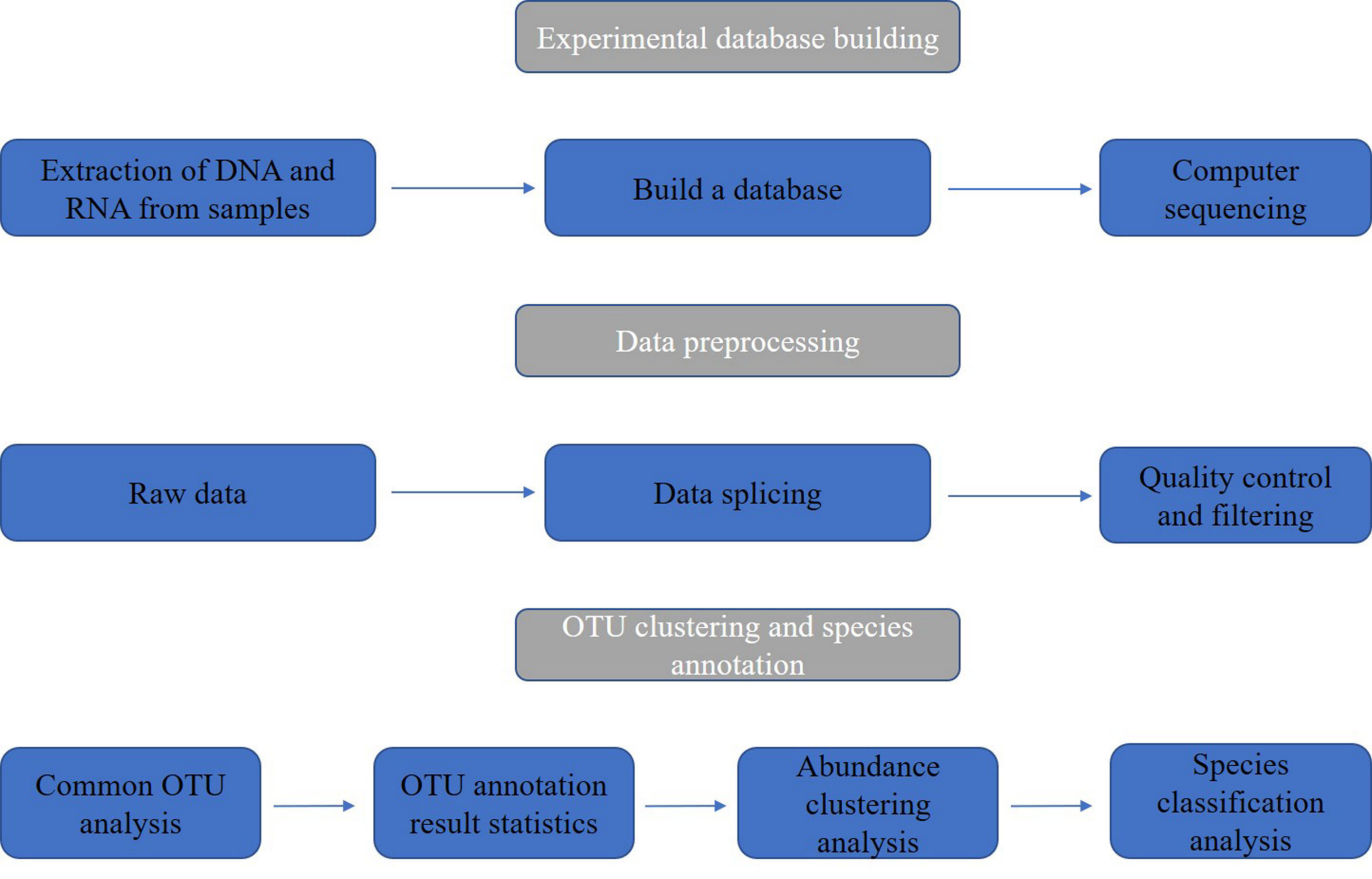
Sample and data processing flowchart of nasopharyngeal swabs from SARS-CoV-2 infected patients.

Metagenomics is an emerging technology developed on the basis of of next-generation sequencing (NGS) technology (Voelkerding et al., 2009). It can study the genomes of microbial populations in specific environments, generating millions to billions of readings in one run, allowing for rapid and accurate analysis of the entire genetic content of clinical or environmental samples (Chiu and Miller, 2019). Compared to traditional microbial research methods, rapid turnover is a major advantage of metagenomic sequencing technology. This study uses NGS as the main method and focuses on the microbial community of nasopharyngeal swabs from patients infected with SARS-CoV-2. Further analysis was carried out by constructing a database, and the sample and data processing flowchart is shown in **Figure 1**.

### Research object

From February 2 through March 20, 2020, we collected samples of oropharynx swabs from SARS-CoV-2 infected-confirmed patients in the Public Health and Clinical Center of Chengdu, China. There was no death in all the cases. In this study, we always observe the privacy rights of human subjects, and all the individuals had given informed consent before enrollment to this study, and samples were obtained.

### Nucleic acid preparation and construction of metagenomic libraries

Total RNA was extracted by TRIzol LS Reagent (ambion^®^, Thermo Fisher Scientific, USA). Reverse Transcription-Polymerase Chain Reaction (RT-PCR) was applied using cDNA Synthesis Kit (Transcriptor First Strand, Roche, Inc., German), and afterward, we constructed a library by Universal V6 RNA-seq library Prep Kit (VAHTS^®^, Vazyme, China) for sequencing.

We extracted total DNA in tested specimens using a TIANamp Swab DNA kit (TIANGEN^®^, TIANGEN, China) and constructed a metagenomic library using KAPA HyperPlus Kit (KAPA, Roche, Inc., German) with dual-indexed Adapters. The DNA was fragmented to approximately 250 bp by the enzyme at 37°C for 20 min. After end repair and A-tailing, adapter ligation, post-ligation Cleanup, library amplification, and post-amplification Cleanup, the library was constructed. Agilent DNA 1000 kit (Agilent, Agilent Technology, Inc., USA) was used for library quality control.

### Sequencing and bioinformatics analysis

The qualified DNA library was sent to the Novogene company for high-throughput sequencing with Illumina Hiseq 4000 (Illumina, USA), and all raw data were trimmed to remove adapter sequence, low-quality reads (bases with a quality value of < 5 accounts for > 50% of the total reads) and duplicate reads. The threshold for the minimum number of reads required to identify microbes is 20. After filtering out the human genome and plasmid genome, microbiological information is obtained. Based on the Krona algorithm, homology comparisons with reference sequences of bacteria, fungi, parasites, and viruses are carried out to identify the classification of microorganisms and draw a complete picture of species composition (Ondov et al., 2011; Wood and Salzberg, 2014). Finally, Heatmaps were generated for non-scaled, non-normalized titer data using a Pearson distance function with average linkage clustering using the program Heml (version 1.0), and statistics features in the study were calculated by IBM SPSS Statistical 21.

## Results

We studied 56 specimens tested for SARS-CoV-2 and other pathogens from 42 patients. All of the samples were detected with SARS-CoV-2. Our study obtained 308.6 GB of data (https://www.ncbi.nlm.nih.gov/sra/?term=SRP273396). After homology comparison, we spotted dangerous respiratory pathogens such as *Mycoplasma pneumoniae, Klebsiella pneumoniae, Legionella pneumophila, Streptococcus pneumoniae, Staphylococcus aureus, Haemophilus influenzae and Human gammaherpesvirus 4*.

### Clinical information

Most of the 42 patients exhibited symptoms of fever and cough, while 5 patients were asymptomatic carriers. As shown in **Table 1**, the majority are middle-aged and elderly, with an average age of approximately 51 years and a female population of 17 (40%). Twenty-one patients were accompanied with basic diseases such as diabetes and hypertension. Among them, a total of 11 (21%) patients had a history of antibiotic use.

**Table 1:** Basic information statistics of patients with COVID-19.

Basic information	Symptomatic (n=37)	Asymptomatic (n=5)	Total (n=42)
Sex			
male	23 (55)	2 (5)	25 (60)
female	14 (33)	3 (7)	17 (40)
Age			
x ± s, years	50.24±15.88	54.00±17.89	50.69±15.94
Underlying disease			
Yes	19 (45)	2 (5)	21 (50)
No	18 (43)	3 (7)	21 (50)
Antibiotic			
Yes	10 (24)	1 (2)	11 (26)
No	27 (64)	4 (10)	31 (74)

Data is expressed as n (%) unless otherwise stated.

### Analysis of species diversity of samples

The results of pathogen analysis except SARS-CoV-2 showed that the respiratory tract microorganisms of 56 samples were mainly bacteria and viruses, and there were relatively many bacterial pathogens. The most common bacteria were *Klebsiella pneumoniae, Legionella pneumophila, Streptococcus pneumoniae, Staphylococcus aureus, Haemophilus influenza*e, and Bordetella bronchiseptica, and the positive rate was 100% (**Table 2**).

**Table 2:** Proportions of Specimens Positive for Non–SARS-CoV-2 Respiratory Pathogens.

Respiratory Pathogens	Proportion for specific respiratory pathogen (percentage)^a^
*Mycoplasma pneumoniae*	35/56 (62.50%)
*Klebsiella pneumoniae*	56/56 (100%)
*Legionella pneumophila*	56/56 (100%)
*Streptococcus pneumoniae*	56/56 (100%)
*Staphylococcus aureus*	56/56 (100%)
*Haemophilus influenzae*	56/56 (100%)
*Bordetella bronchiseptica*	56/56 (100%)
*B. parapertussis*	45/56 (80.36%)
*B. pertussis*	38/56 (67.86%)
*Moraxella catarrhalis*	53/56 (94.64%)
Rhinovirus/enterovirus	1/56 (1.79%)
Adenovirus	24/56 (42.86%)
Human Respiratory Syncytial Virus	3/56(5.36%)
Human respirovirus 3	10/56 (17.86%)
Metapneumovirus	1/56 (1.79%)
Human alphaherpesvirus 3	2/56 (3.57%)
Human gammaherpesvirus 4	30/56 (53.57%)
Human betaherpesvirus 5	20/56 (35.71%)
Human betaherpesvirus 6	13/56 (23.21%)
Influenza A virus	2/56 (3.57%)
Human associated gemykibivirus 2	6/56 (10.71%)

a Positive results for non–SARS-CoV-2 pathogens may, in some cases, represent the detection of residual virus in resolved cases rather than clinical co-infection as such, and the proportions of respiratory pathogens are calculated concerning numbers of tested appropriate samples

### Analysis of community composition of samples

The community composition of oropharyngeal swab samples from patients with SARS-CoV-2 infection was analyzed. The pathogens in COVID-19 patients included bacteria, fungi, and viruses. In this study, the relative abundance sum of all samples was sorted, and the top ten pathogens in each sample were observed. As shown by **Table 3**, the relative abundance of bacteria (top 10 > 1%) and viruses (top 4 < 0.1%) are relatively high in oral and pharyngeal swab samples of patients with SARS-CoV-2 infection.

**Table 3:** The principal bacteria, fungi, and viruses existed in the tested COVID-19 patients.

Pathogen	The percentage of pathogens’ reads in total microbe sequences, mean (range)^a^
Bacteria	
* Prevotella melaninogenica*	10.5025% (0.7247%-47.3168%)
* Veillonella parvula*	4.3350% (1.1662%-31.8871%)
* Veillonella dispar*	6.9966% (0.4530%-23.9620%)
* Prevotella intermedia*	2.4605% (0.8188%-6.5189%)
* Cutibacterium acnes*	3.5464% (0.4655%-9.9811%)
* Prevotella jejuni*	6.7800% (0.9270%-15.5768%)
* Rothia mucilaginosa*	4.6652% (1.3835%-18.1679%)
* Bradyrhizobiaceae bacterium SG-6C*	3.9947% (0.5253%-21.9056%)
* Fusobacterium periodonticum*	3.1706% (1.4863%-6.2479%)
* Streptococcus mitis*	2.6718% (1.2473%-7.5728%)
Fungi	
* Botrytis cinerea*	0.1703% (0.0045%-3.0243%)
* Aspergillus oryzae*	0.0612% (0.0044%-0.5264%)
* Yarrowia lipolytica*	0.1705% (0.0061%-0.7637%)
* Colletotrichum higginsianum*	0.0437% (0.0022%-0.2592%)
* Komagataella phaffii*	0.2207% (0.0062%-1.0449%)
* Malassezia restricta*	1.2193% (0.0078%-33.3478%)
* Thermothielavioides terrestris*	0.0318% (0.0020%-0.1902%)
* Neurospora crassa*	0.0238% (0.0020%-0.1367%)
* Candida albicans*	0.0799% (0.0031%-1.2912%)
* Candida dubliniensis*	0.0626% (0.0048%-0.2465%)
Viruses	
Avian leukosis virus	0.0286% (0.0011%-0.2061%)
Huangpi Tick Virus 1	0.0073% (0.00022%-0.0203%)
Rous sarcoma virus	0.0082% (0.0013%-0.0226%)
Human gammaherpesvirus 4	0.0050% (0.0010%-0.0128%)

a The abundance included the top ten pathogens in each sample and the corresponding reads.

**Figure 2:**
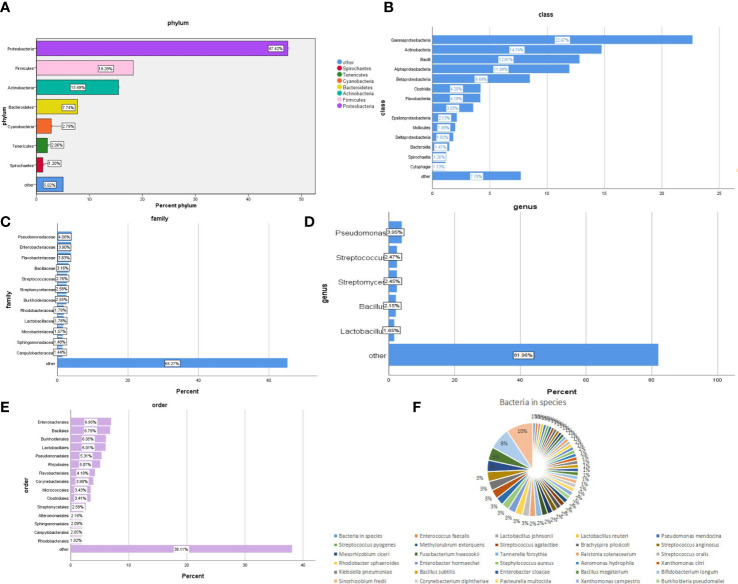
Composition of the bacterial community in patients with SARS-CoV-2 infection. The bacterial communities of all samples in the phylum, class, order, family, genus, and species are **(A-F)** respectively.

The bacterial community composition of all samples in the phylum, class, order, family, genus, and species is shown in **Figure 2**. The largest proportion of phylum bacteria is *Proteobacteria* (47.6%), *Firmicutes* (18.3%), and *Actinobacteria* (15.5%). The largest proportion of species of bacteria is *Escherichia coli* (9.5%), *Salmonella enterica* (7.6%), and *Bacillus cereus* (4.6%).

**Figure 3:**
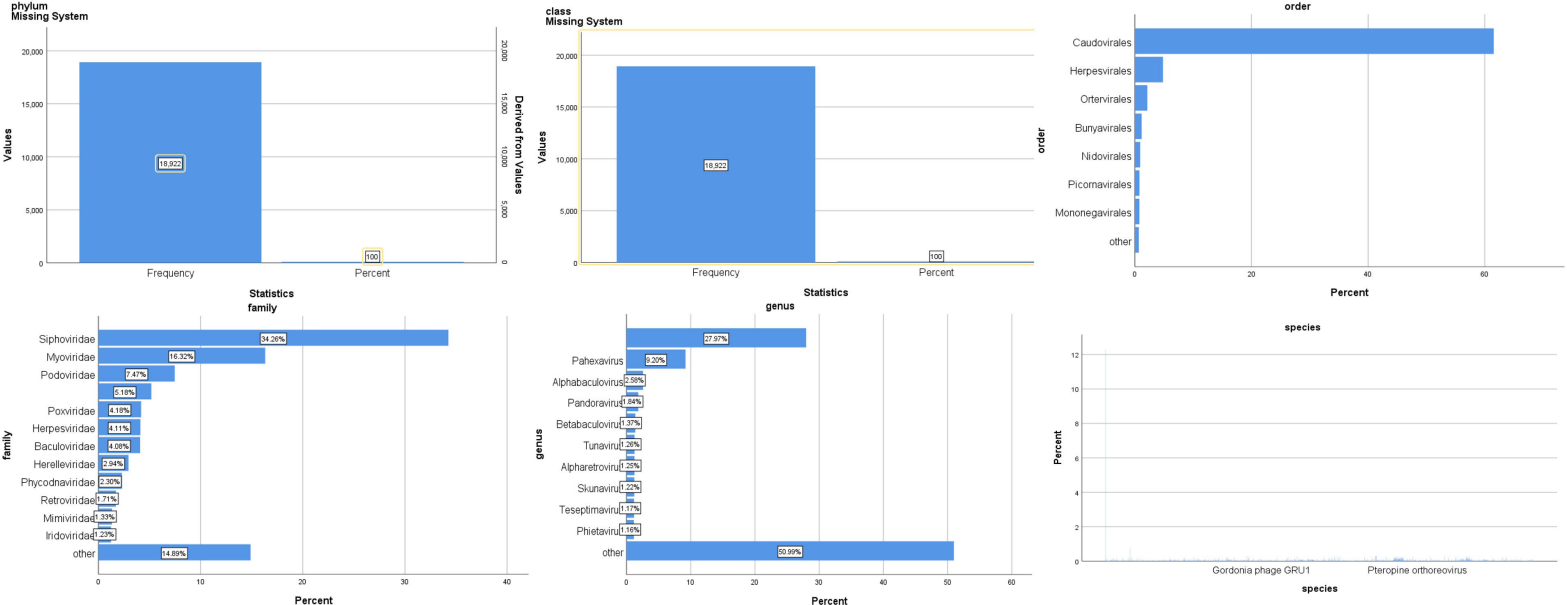
Composition of the virus community in patients with SARS-CoV-2 infection.

The virus community composition of all samples is shown in **Figure 3**. Phylum in the virus is all of *Negarnaviricota* (100%). The largest proportion of viral species is *Avian leukosis virus* (16%), *Pandoravirus inopinatum* (9.7%), and *Pandoravirus neocaledonia* (9.7%).

**Figure 4:**
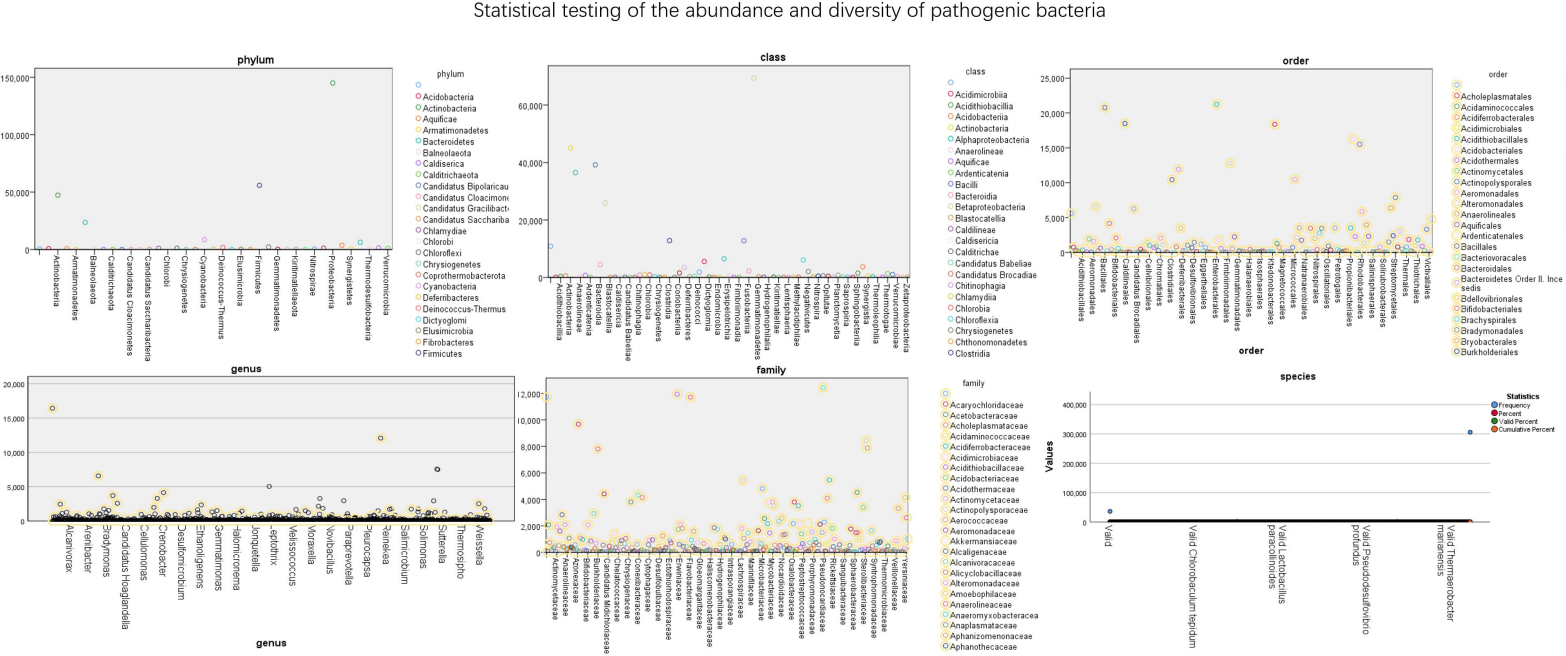
Statistical testing of the abundance and diversity of pathogenic bacteria.

The abundance of bacteria in different dimensions of nasopharyngeal swabs is shown in **Figure 4**. It further quantifies the specific number of pathogens in these 56 test samples. The numerical values in the figure represent the abundance of pathogens. From the figure, it can be seen that *Proteobacteria* has the highest proportion among different bacterial groups, followed by *Gammaproteobacteria*.

**Figure 5:**
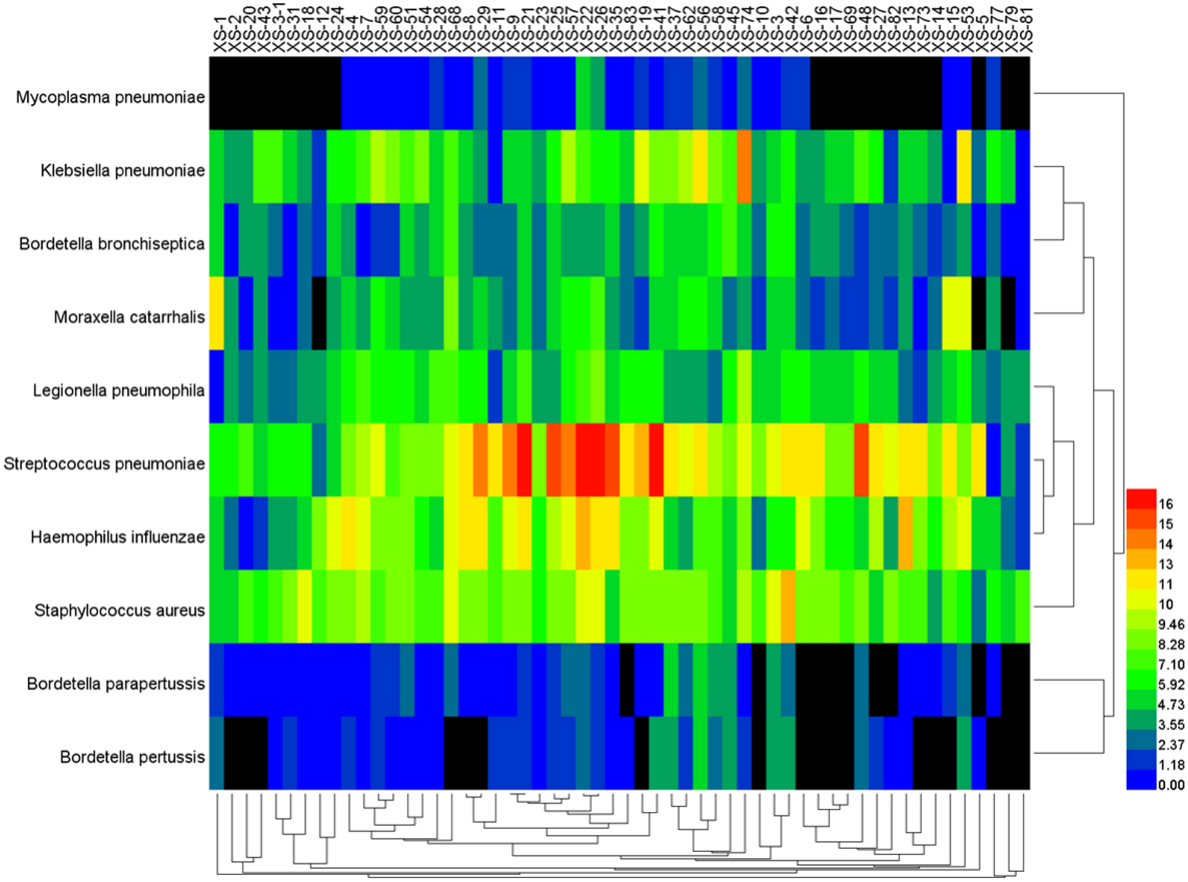
Heat map of the co-infected bacteria abundance from SARS-CoV-2 infected patients.

**Figure 6:**
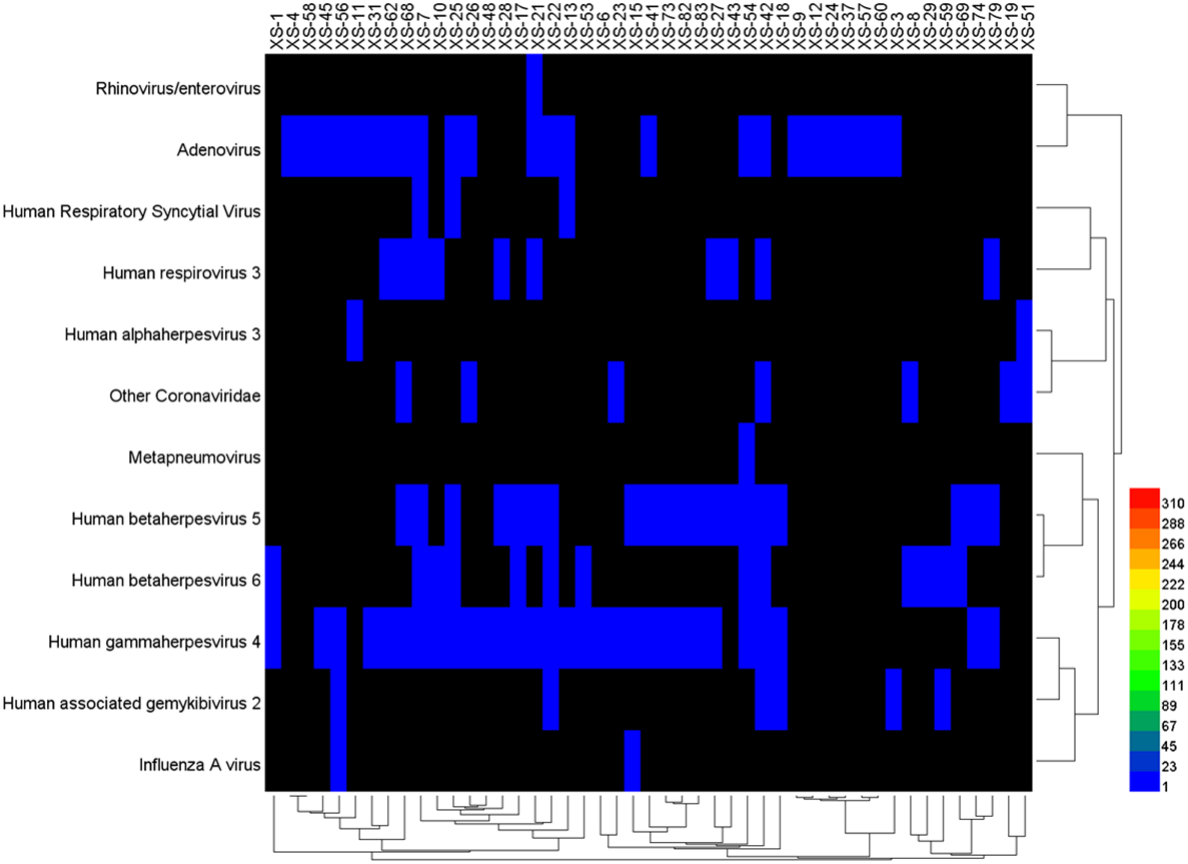
Heat map of the co-infected virus abundance from Sars-CoV-2 infected patients.

The data of reads from the analytical outcome of Krona was integrated and then drew heat map that directly demonstrates an abundance of information on respiratory pathogens about bacteria (**Figure 5**) and viruses (**Figure 6**). In our study, the abundance of the above bacteria is mostly more than 1000, and the abundance of these viruses was generally smaller than 500. As shown by **Figure 4**, the relative abundance of *Streptococcus pneumoniae, Haemophilus influenzae, Staphylococcus aureus*, and *Klebsiella pneumoniae* was higher in the main co-infected bacterial communities in patients with SARS-CoV-2 infection. Among the main co-infected virus communities in patients with SARS-CoV-2 infection, the relative abundance of *Human gammaherpesvirus 4* was the highest.

### Difference analysis

**Figure 7:**
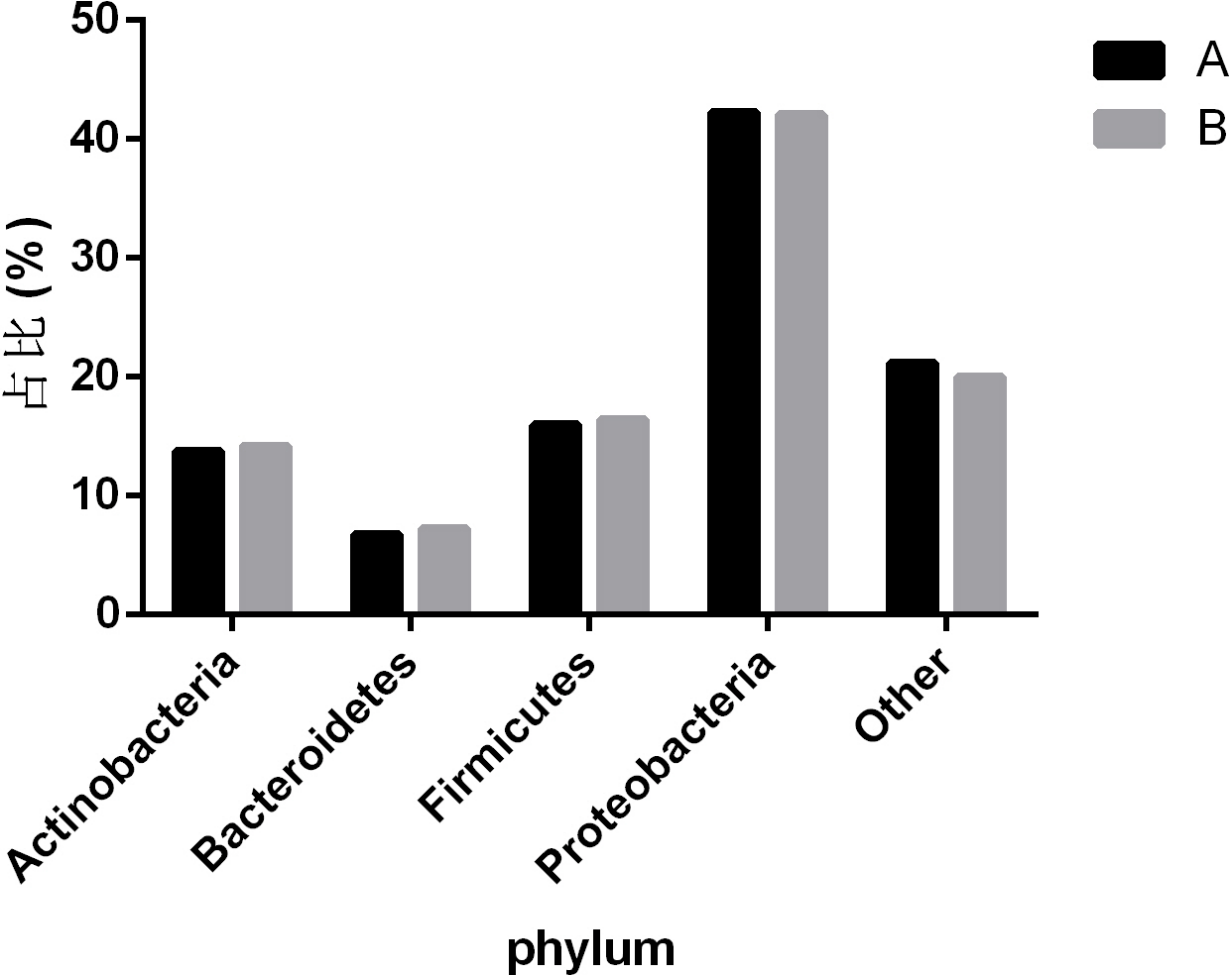
Comparison of nasopharyngeal microbiota in COVID-19 patients with or without antibiotic. A refers to the group of COVID-19 patients without antibiotics, and B refers to the group of COVID-19 patients with antibiotics. The figure mainly shows bacteria with a proportion greater than 1%.

**Figure 8:**
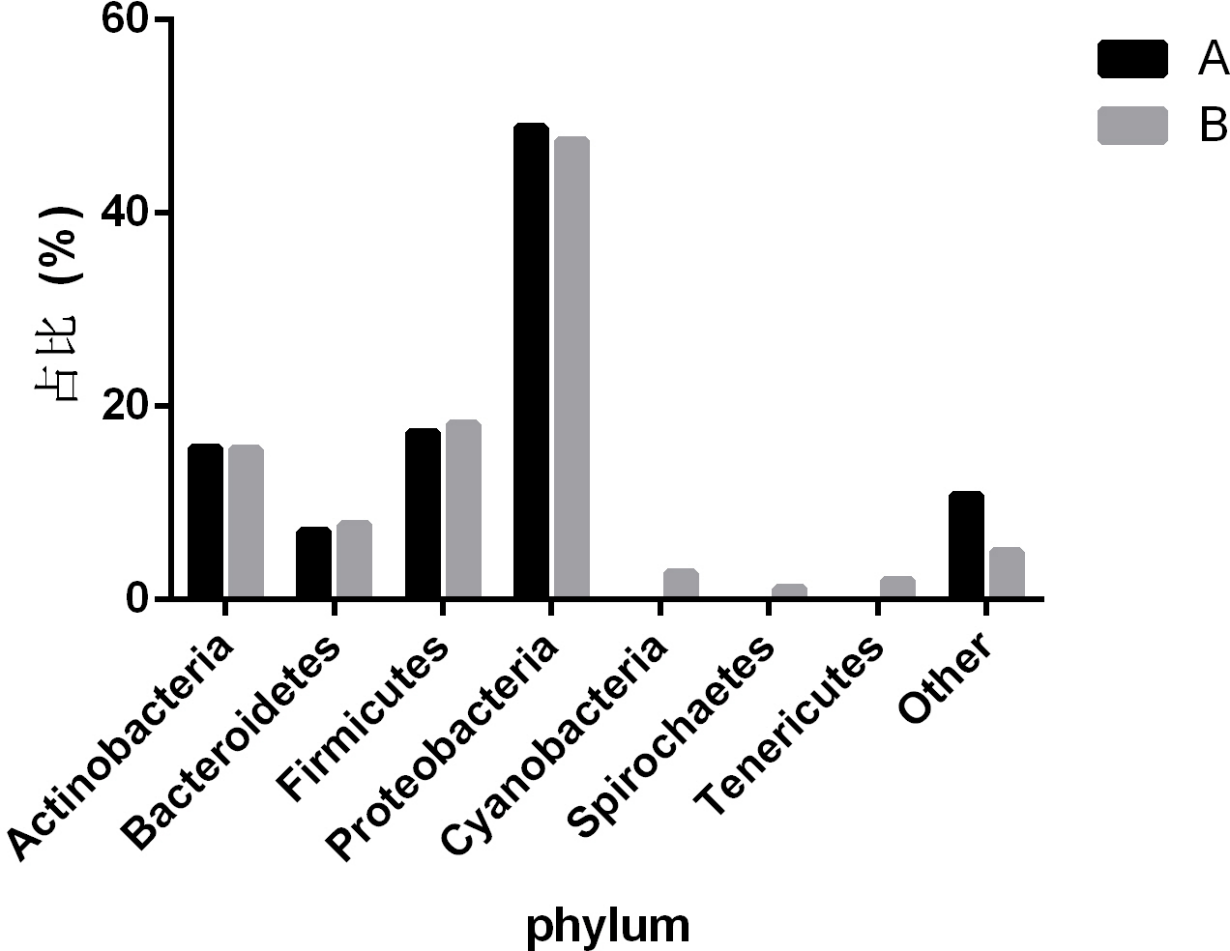
Comparison of nasopharyngeal microbiota between symptomatic and asymptomatic COVID-19 patients. A refers to the asymptomatic COVID-19 patient group, and B refers to the symptomatic COVID-19 patient group. The figure mainly shows bacteria with a proportion greater than 1%.

To observe the differences in nasopharyngeal microbiota between patients with and without symptomatic symptoms and the use of antibiotics, the samples were divided into groups with and without antibiotic use, as well as symptomatic and asymptomatic groups. Plot the abundance data of each species at the phylum level into a column chart. We found that, consistent with the results of the combined analysis of all samples, the nasopharyngeal microbiota of patients with or without antibiotic use and without symptoms were mainly *Proteobacteria*, *Firmicutes*, and *Actinobacteria*. The comparison of differences shows that the main microflora in the nasopharynx of patients with COVID-19 infection in the antibiotic group was not significantly changed compared with those in the non antibiotic group (**Figure 7**). In the other group of comparison, we found that *Cyanobacteria*, *Spirochaetes* and *Tenericites* were significantly higher in symptomatic infected people than in asymptomatic COVID-19 infected people (**Figure 8**).

## Discussion

The current COVID-19 pandemic has entered a stable period under the effective control of government departments and has gradually become a “normal” existence similar to influenza viruses. However, deaths after COVID-19 infection still occur from time to time, and states should take appropriate measures to reduce the number of hospitalizations and deaths. Coinfections and superinfections are common in respiratory viral infections (McArdle et al., 2018; Paget and Trottein, 2019). It has been reported that coinfection caused by opportunistic pathogens in the host may significantly inhibit the immune system and adversely affect the prognosis (Li and Zhou, 2013), which may increase the mortality rate of patients infected with the virus (Beadling and Slifka, 2004; Metzger and Sun, 2013). In addition to coronaviruses, infections caused by opportunistic pathogens can also increase the burden on the host (Bengoechea and Bamford, 2020).

In this study, the frequently identified bacteria among COVID-19 patients were *K. pneumoniae*, *S. pneumoniae*, and *S. aureus*, which was in line with other studies. Consistent with the results of other studies, SARS-CoV-2 has a threatening coinfection with *K. pneumoniae*, *L. pneumophila*, and *S. pneumoniae* (Zhu et al., 2020; Musuuza et al., 2021). Despite low rates of coinfection bacteria and fungus among COVID-19 patients (Rawson et al., 2020), the detrimental impact of coinfection is a delicate issue in public health. That is because the knowledge of SARS-CoV-2 was yet to be fully understood, and relevant research revealed potential detriment, such as SARS-CoV-2 associated infection in patients with TB and various bacteria were identified in various samples among SARS patients (Tan et al., 2005; Motta et al., 2020; Tadolini et al., 2020). Some investigators also speculated that some patients die from bacterial coinfection rather than the virus (Mirzaei et al., 2020).

Some identified bacteria in our study, mostly conditional pathogens, are presented below. They might become pathogenic when the immune function is weakened (Zhu et al., 2020). *K. pneumoniae* and *H. influenza* were some of the most frequent co-infecting pathogens (Lansbury et al., 2020), and these bacteria were found similarly in our study. *K. pneumoniae* is one of the important pathogens for clinical isolation and nosocomial infection, which exists in the human body’s upper respiratory tract and intestines. Under the condition of dysbacteria by abusing antibiotics or impaired function of immunity, *K. pneumoniae* might cause Klebsiella pneumonia with the symptom of acute onset, high fever, cough, and purulent sputum.

Among Pathogeniccoccus, *S. pneumoniae* is one of the severest pathogens in pathogenicity and a frequent cause of superinfection in the respiratory tract (Klein et al., 2016). Through pneumococcal hemolysin and capsule, *S. pneumoniae* can cause lobar pneumonia, cephalomeningitis, bronchitis, and so forth (Musher, 1992). Pneumonia caused by *S. pneumoniae* is a sudden onset, fever, chills, severe pectoralgia, and rusty sputum. Because *S. pneumoniae* is sensitive to various antibiotics, penicillin G is the preferred remedy.

*S. aureus* is the most common pathogenic bacterium in human suppurative infections. *S. aureus* can cause local pyogenic infection, also causing pneumonia, pseudomembranous enteritis, pericarditis, and sepsis. The pathogenicity depends on the toxins and invasive enzymes. *Staphylococcal pneumonia* is characterized by repeated chills, tissue necrosis with abscess formation, and pulmonary cysts (mainly in infants); empyema and purulent sputum are more common (Cheung et al., 2021). Studies have shown that the rate of *S. aureus* infection in COVID-19 patients is significantly higher than in uninfected SARS-CoV-2 patients (Singh et al., 2021).

*Influenza A virus* (IAV) is highly pathogenic and mutates readily. The respiratory illnesses caused by IAV usually occur in winter and have been associated with disease outbreaks in several countries (Lafond et al., 2016). There have been many reports about infection of SARS-CoV-2 and IAV (Alshaikh et al., 2022). In a study by Xiang et al (Mirzaei et al., 2020), compared with SARS-CoV-2 infection, many patients co-infected with SARS-CoV-2, and IAV had severe dyspnea on admission. Coinfection of SARS-CoV-2 and IAV may develop more severe clinical conditions.

*Human herpesvirus 4* (HHV4), or EB Virus (EBV), is the causative agent of infectious mononucleosis and is associated with nasopharyngeal carcinoma and childhood lymphoma. Infectious mononucleosis displays as fever, enlargement of lymph nodes, pharyngalgia, and hepatosplenomegaly (Nowalk and Green, 2016). The early treatment of ganciclovir and interferon can relieve symptoms, but it is noneffective for latent infection of EBV. EBV infection is widespread in the population. We also detected other viruses, such as Rhinovirus/enterovirus, Adenovirus, Human Respiratory Syncytial Virus, and Human retrovirus, consistent with previous studies (Kim et al., 2020; Lin et al., 2020). Studies have shown that the coinfection rate of EBV in SARS-CoV-2 positive samples is higher than that of other respiratory viruses, which is related to the immune status of the host (Singh et al., 2021).

Apart from these, some pathogenetic pathogens were tested by metagenomics sequencing, and these causative agents account for a large part of total pathogens. For instance, bacteria such as *P. melaninogenica*, *R. mucilaginosa*, *S. mitis* and fungi such as *C. albicans*, *C. dubliniensis* are opportunistic pathogens, which are fatal for critically ill patients with frequently taking antibacterial agents, impaired immunity, and long-term hospitalization. If these pathogens are found clinically, they will be a deadly threat to COVID-19 patients with severe illness.

Our research has linked the presence or absence of symptoms of COVID-19 infected people with the ecological imbalance of nasopharynx microorganisms, including the changes in microbial abundance and specific taxon abundance. It is worth noting that *Cyanobacteria*, *Spirochaetes*, and *Tenericutes* have a relatively high abundance in symptomatic patients, while only a few are in asymptomatic patients. This indicates that the presence of *Cyanobacteria*, *Spirochaetes* and *Tenericutes* may be related to the symptoms of patients with COVID-19. *Cyanobacteria* exists in all aquatic ecosystem of the earth, and can enter the human nasopharynx through the mucosal surface. It can cause pneumonia and liver damage by producing a variety of secondary metabolites (Buratti et al., 2017). *Spirochaetes* is a kind of bacteria with very special characteristics, which can move by virtue of the twisting movement generated by its unique “axoneme”. *Borrelia burgdorferi* is one of the more important members (Paster and Dewhirst, 2000). The presence of SARS-CoV-2 combined with *Spirochaetes* may be one of the reasons for relatively severe symptoms. The study proved that there was a significant correlation between the serum level of anti *Spirochaetes* antibodies observed in the same individual and the severity of COVID-19, and the serum level of anti *Spirochaetes* antibodies was closely related to the severity of COVID-19 and the risk of hospitalization (Szewczyk-Dąbrowska et al., 2022). *Tenericutes* have a single membrane and lack a cell wall, and can form a close relationship with humans in a symbiotic or parasitic state (Razin et al., 1998). *Mycoplasma*, as one of the important branches of *Tenericutes*, may also be the cause of the difference in symptoms among COVID-19 patients. A study reported differences in the microbial structure and composition of the nasopharynx between SARS-CoV-2 infected and uninfected pregnant women. The results showed that compared to uninfected individuals, SARS-CoV-2 infected individuals showed changes in their nasopharyngeal microbiota, with a higher relative abundance of *Tenericutes* (Crovetto et al., 2022).

In this study, the pathogens in COVID-19 patients were detected by metagenome sequencing and analysis, providing data support for the rapid identification of coexisting pathogens in COVID-19 patients (including potential patients). Besides, the outcome of this method is quite instructive for the treatment of subsequent COVID-19 patients. The situation that secondary bacterial infections lead to severer clinical outcomes should arouse increasing public concern. To prevent secondary infection caused by coexisting pathogens, it is important to comprehend how viruses and bacteria interact, enabling the development of more effective diagnostic and treatment approaches. However, this study is limited to a single district. The metagenomic analysis has some limitation because of the given sample size and spatiotemporal variation in viral epidemiology.

## Future work

In the future, researchers will find out which pathogens are easy to induce related geriatric diseases in the elderly according to the analysis of coexisting pathogens in the positive patients, and the experimental results will guide clinical adjustment of COVID-19 treatment and medication programs for elderly patients.

## Ethical approval

In this study, we always observe the privacy rights of human subjects, and all the individuals had given informed consent before enrollment to this study, and samples were obtained.

## Data availability statement

The data presented in the study are deposited in the NCBI repository, accession number SRP273396.

## Ethics statement

Written informed consent was obtained from the individual(s) for the publication of any potentially identifiable images or data included in this article.

## Author contributions

All author contributions are listed in order of author. Study concept and design: ZG, LY and LC. MY was responsible for all the revisions and data additions in the manuscript. Acquisition and data analysis: YLi, YLan, RT, YH, GL and YLiu. Drafting of the manuscript: LY, HY, LJ, YZ, ZF and YB. ML, MH, and SD had full access to all the data in the study and took responsibility for the integrity of the data and the accuracy of the data analysis. All authors contributed to data interpretation and manuscript completion.

## References

[B1] Alshaikh F. S. Godman B. Sindi O. N. Seaton R. A. Kurdi A. (2022). Prevalence of bacterial coinfection and patterns of antibiotics prescribing in patients with COVID-19: a systematic review and meta-analysis. PloS One 17, e0272375. doi: 10.1371/journal.pone.0272375 35913964 PMC9342726

[B2] Arashiro T. Nakamura S. Asami T. Mikuni H. Fujiwara E. Sakamoto S.. (2020). SARS-CoV-2 and legionella co-infection in a person returning from a Nile cruise. J. Travel Med. 27 (3), taaa053. doi: 10.1093/jtm/taaa053 32297939 PMC7184515

[B3] WHO (2020)Coronavirus disease (COVID-19) dashboard. In: World health organization. Available at: https://covid19.who.int/ (Accessed December 2022, 2022).

[B4] Beadling C. Slifka M. K. (2004). How do viral infections predispose patients to bacterial infections? Curr. Opin. Infect. Dis. 17, 185–191. doi: 10.1097/00001432-200406000-00003 15166819

[B5] Bengoechea J. A. Bamford C. G. (2020). SARS-CoV-2, bacterial co-infections, and AMR: the deadly trio in COVID-19? EMBO Mol. Med. 12, e12560. doi: 10.15252/emmm.202012560 32453917 PMC7283846

[B6] Buratti F. M. Manganelli M. Vichi S. Stefanelli M. Scardala S. Testai E.. (2017). Cyanotoxins: producing organisms, occurrence, toxicity, mechanism of action and human health toxicological risk evaluation. Arch. Toxicol. 91, 1049–1130. doi: 10.1007/s00204-016-1913-6 28110405

[B7] Chen Y. Klein S. L. Garibaldi B. T. Li H. Wu C. Osevala N. M.. (2021). Aging in COVID-19: vulnerability, immunity and intervention. Ageing Res. Rev. 65, 101205. doi: 10.1016/j.arr.2020.101205 33137510 PMC7604159

[B8] Cheung G. Y. C. Bae J. S. Otto M. (2021). Pathogenicity and virulence of staphylococcus aureus. Virulence 12, 547–569. doi: 10.1080/21505594.2021.1878688 33522395 PMC7872022

[B9] Chiu C. Y. Miller S. A. (2019). Clinical metagenomics. Nat. Rev. Genet. 20, 341–355. doi: 10.1038/s41576-019-0113-7 30918369 PMC6858796

[B10] Crovetto F. Selma-Royo M. Crispi F. Carbonetto B. Pascal R. Larroya M.. (2022). Nasopharyngeal microbiota profiling of pregnant women with SARS-CoV-2 infection. Sci. Rep. 12, 13404. doi: 10.1038/s41598-022-17542-z 35927569 PMC9352760

[B11] de Steenhuijsen Piters W. A. Huijskens E. G. Wyllie A. L. Biesbroek G. van den Bergh M. R. Veenhoven R. H.. (2016). Dysbiosis of upper respiratory tract microbiota in elderly pneumonia patients. Isme J. 10, 97–108. doi: 10.1038/ismej.2015.99 26151645 PMC4681870

[B12] Gao Z. Kang Y. Yu J. Ren L. (2014). Human pharyngeal microbiome may play a protective role in respiratory tract infections. Genomics Proteomics Bioinf. 12, 144–150. doi: 10.1016/j.gpb.2014.06.001 PMC4411333 24953866

[B13] Kim D. Quinn J. Pinsky B. Shah N. H. Brown I. (2020). Rates of Co-infection between SARS-CoV-2 and other respiratory pathogens. Jama 323, 2085–2086. doi: 10.1001/jama.2020.6266 32293646 PMC7160748

[B14] Klein E. Y. Monteforte B. Gupta A. Jiang W. May L. Hsieh Y. H.. (2016). The frequency of influenza and bacterial coinfection: a systematic review and meta-analysis. Influenza Other Respir. Viruses 10, 394–403. doi: 10.1111/irv.12398 27232677 PMC4947938

[B15] Lafond K. E. Nair H. Rasooly M. H. Valente F. Booy R. Rahman M.. (2016). Global role and burden of influenza in pediatric respiratory hospitalizations, 1982-2012: a systematic analysis. PloS Med. 13, e1001977. doi: 10.1371/journal.pmed.1001977 27011229 PMC4807087

[B16] Lansbury L. Lim B. Baskaran V. Lim W. S. (2020). Co-Infections in people with COVID-19: a systematic review and meta-analysis. J. Infect. 81, 266–275. doi: 10.1016/j.jinf.2020.05.046 32473235 PMC7255350

[B17] Li X. X. Zhou X. N. (2013). Co-Infection of tuberculosis and parasitic diseases in humans: a systematic review. Parasit Vectors 6, 79. doi: 10.1186/1756-3305-6-79 23522098 PMC3614457

[B18] Lin D. Liu L. Zhang M. Hu Y. Yang Q. Guo J.. (2020). Co-Infections of SARS-CoV-2 with multiple common respiratory pathogens in infected patients. Sci. China Life Sci. 63, 606–609. doi: 10.1007/s11427-020-1668-5 32170625 PMC7089461

[B19] McArdle A. J. Turkova A. Cunnington A. J. (2018). When do co-infections matter? Curr. Opin. Infect. Dis. 31, 209–215. doi: 10.1097/QCO.0000000000000447 29698255 PMC6037283

[B20] Metzger D. W. Sun K. (2013). Immune dysfunction and bacterial coinfections following influenza. J. Immunol. 191, 2047–2052. doi: 10.4049/jimmunol.1301152 23964104 PMC3760235

[B21] Mirzaei R. Goodarzi P. Asadi M. Soltani A. Aljanabi H. A. A. Jeda A. S.. (2020). Bacterial co-infections with SARS-CoV-2. IUBMB Life 72, 2097–2111. doi: 10.1002/iub.2356 32770825 PMC7436231

[B22] Morris D. E. Cleary D. W. Clarke S. C. (2017). Secondary bacterial infections associated with influenza pandemics. Front. Microbiol. 8, 1041. doi: 10.3389/fmicb.2017.01041 28690590 PMC5481322

[B23] Motta I. Centis R. D'Ambrosio L. García-García J. M. Goletti D. Gualano G.. (2020). Tuberculosis, COVID-19 and migrants: preliminary analysis of deaths occurring in 69 patients from two cohorts. Pulmonology 26, 233–240. doi: 10.1016/j.pulmoe.2020.05.002 32411943 PMC7221402

[B24] Musher D. M. (1992). Infections caused by streptococcus pneumoniae: clinical spectrum, pathogenesis, immunity, and treatment. Clin. Infect. Dis. 14, 801–807. doi: 10.1093/clinids/14.4.801 1576274

[B25] Musuuza J. S. Watson L. Parmasad V. Putman-Buehler N. Christensen L. Safdar N. (2021). Prevalence and outcomes of co-infection and superinfection with SARS-CoV-2 and other pathogens: a systematic review and meta-analysis. PloS One 16, e0251170. doi: 10.1371/journal.pone.0251170 33956882 PMC8101968

[B26] Nowalk A. Green M. (2016). Epstein-Barr Virus. Microbiol. Spectr. 4, 127–134. doi: 10.1128/9781555819040.ch5 27337443

[B27] Ondov B. D. Bergman N. H. Phillippy A. M. (2011). Interactive metagenomic visualization in a web browser. BMC Bioinf. 12, 385. doi: 10.1186/1471-2105-12-385 PMC3190407 21961884

[B28] Paget C. Trottein F. (2019). Mechanisms of bacterial superinfection post-influenza: a role for unconventional T cells. Front. Immunol. 10, 336. doi: 10.3389/fimmu.2019.00336 30881357 PMC6405625

[B29] Paster B. J. Dewhirst F. E. (2000). Phylogenetic foundation of spirochetes. J. Mol. Microbiol. Biotechnol. 2, 341–344. 11075904

[B30] Rawson T. M. Moore L. S. P. Zhu N. Ranganathan N. Skolimowska K. Gilchrist M.. (2020). Bacterial and fungal coinfection in individuals with coronavirus: a rapid review to support COVID-19 antimicrobial prescribing. Clin. Infect. Dis. 71, 2459–2468. doi: 10.1093/cid/ciaa530 32358954 PMC7197596

[B31] Razin S. Yogev D. Naot Y. (1998). Molecular biology and pathogenicity of mycoplasmas. Microbiol. Mol. Biol. Rev. 62, 1094–1156. doi: 10.1128/MMBR.62.4.1094-1156.1998 9841667 PMC98941

[B32] Rothe K. Feihl S. Schneider J. Wallnöfer F. Wurst M. Lukas M.. (2021). Rates of bacterial co-infections and antimicrobial use in COVID-19 patients: a retrospective cohort study in light of antibiotic stewardship. Eur. J. Clin. Microbiol. Infect. Dis. 40, 859–869. doi: 10.1007/s10096-020-04063-8 33140176 PMC7605734

[B33] Singh V. Upadhyay P. Reddy J. Granger J. (2021). SARS-CoV-2 respiratory co-infections: incidence of viral and bacterial co-pathogens. Int. J. Infect. Dis. 105, 617–620. doi: 10.1016/j.ijid.2021.02.087 33640570 PMC7905386

[B34] Stasi C. Fallani S. Voller F. Silvestri C. (2020). Treatment for COVID-19: an overview. Eur. J. Pharmacol. 889, 173644. doi: 10.1016/j.ejphar.2020.173644 33053381 PMC7548059

[B35] Szewczyk-Dąbrowska A. Budziar W. Harhala M. Baniecki K. Pikies A. Jędruchniewicz N.. (2022). Dąbrowska, correlation between COVID-19 severity and previous exposure of patients to borrelia spp. Sci. Rep. 12, 15944. doi: 10.1038/s41598-022-20202-x 36153350 PMC9509370

[B36] Tadolini M. Codecasa L. R. García-García J. M. Blanc F. X. Borisov S. Alffenaar J. W.. (2020). Sequelae and COVID-19 co-infection: first cohort of 49 cases. Eur. Respir. J. 56 (1), 2001398. doi: 10.1183/13993003.01398 32457198 PMC7251245

[B37] Tan F. L. Loo W. L. Tan S. G. Wong C. Y. Tan Y. M. (2005). Severe acute respiratory syndrome in surgical patients: a diagnostic dilemma. ANZ J. Surg. 75, 21–26. doi: 10.1111/j.1445-2197.2005.03285.x 15740510

[B38] Touzard-Romo F. Tapé C. Lonks J. R. (2013). Co-Infection with SARS-CoV-2 and human metapneumovirus. R I Med. J. 103(2020), 75–76. 32192233

[B39] Voelkerding K. V. Dames S. A. Durtschi J. D. (2009). Next-generation sequencing: from basic research to diagnostics. Clin. Chem. 55, 641–658. doi: 10.1373/clinchem.2008.112789 19246620

[B40] Wood D. E. Salzberg S. L. (2014). Kraken: ultrafast metagenomic sequence classification using exact alignments. Genome Biol. 15, R46. doi: 10.1186/gb-2014-15-3-r46 24580807 PMC4053813

[B41] Wu X. Cai Y. Huang X. Yu X. Zhao L. Wang F.. (2020). Co-Infection with SARS-CoV-2 and influenza a virus in patient with pneumonia, China. Emerg. Infect. Dis. 26, 1324–1326. doi: 10.3201/eid2606.200299 32160148 PMC7258479

[B42] Xie J. Wang Q. Xu Y. Zhang T. Chen L. Zuo X.. (2021). Clinical characteristics, laboratory abnormalities and CT findings of COVID-19 patients and risk factors of severe disease: a systematic review and meta-analysis. Ann. Palliat Med. 10, 1928–1949. doi: 10.21037/apm-20-1863 33548996

[B43] Zhu F. Cao Y. Xu S. Zhou M. (2020). Co-Infection of SARS-CoV-2 and HIV in a patient in wuhan city, China. J. Med. Virol. 92, 529–530. doi: 10.1002/jmv.25732 32160316 PMC7228399

[B44] Zhu X. Ge Y. Wu T. Zhao K. Chen Y. Wu B.. (2020). Co-Infection with respiratory pathogens among COVID-2019 cases. Virus Res. 285, 198005. doi: 10.1016/j.virusres.2020.198005 32408156 PMC7213959

